# Testicular Immunity and Its Connection with the Microbiota. Physiological and Clinical Implications in the Light of Personalized Medicine

**DOI:** 10.3390/jpm12081335

**Published:** 2022-08-20

**Authors:** Luigi Santacroce, Ciro Imbimbo, Andrea Ballini, Felice Crocetto, Salvatore Scacco, Stefania Cantore, Erika Di Zazzo, Marica Colella, Emilio Jirillo

**Affiliations:** 1 Interdisciplinary Department of Medicine, Section of Microbiology and Virology, University of Bari “Aldo Moro”, 70124 Bari, Italy; 2 Department of Neuroscience, Reproductive Sciences and Dentistry, School of Medicine, University of Naples “Federico II”, 80131Naples, Italy; 3 Department of Precision Medicine, University of Campania “Luigi Vanvitelli”, 80138 Naples, Italy; 4 Department of Basic Medical Sciences, Neurosciences and Sense Organs, University of Bari “Aldo Moro”, 70124 Bari, Italy; 5 Independent Researcher, Sorriso & Benessere—Ricerca e Clinica, 70129 Bari, Italy; 6 Department of Medicine and Health Sciences “V. Tiberio”, University of Molise, 86100 Campobasso, Italy

**Keywords:** immune privilege, microbiota, male infertility, spermatogenesis, testis, polyphenols, probiotics, personalized medicine

## Abstract

Reproduction is a complex process, which is based on the cooperation between the endocrine–immune system and the microbiota. Testicular immunity is characterized by the so-called immune privilege, a mechanism that avoids autoimmune attacks against proteins expressed by spermatozoa. Testicular microbiota is connected with the gut microbiota, the most prevalent site of commensals inthe body. Both microbiotas take part inthe development of the immune system and protection againstpathogen invasion. Dysbiosis is caused by concurrent pathologies, such as obesity, diabetes, infections and trauma. The substitution of beneficial bacteria with pathogens may lead to destruction of spermatozoa directly or indirectly and, ultimately, to male infertility. Novel therapeutic interventions, i.e., nutritional interventions and supplementation of natural products, such as, probiotics, prebiotics, antioxidants and polyphenols, may lead to the restoration of the otherwise-impaired male reproductive potential, even if experimental and clinical results are not always concordant. In this review, the structure and immune function of the testis will be described with special reference to the blood–testisbarrier. The regulatory role of both the gut and testicular microbiota will be illustrated in health and disease, also emphasizing therapeutic attempts with natural products for the correction of male infertility, in the era of personalized medicine.

## 1. Introduction

The testis is a continuous source of germ cells as the first step of male reproduction, followed by the transport of sperm to the fallopian tube sperm–egg binding sites [1]. In general terms, reproduction is a complex process, which requires a strict collaboration between the endocrine and the immune system. In fact, spermatogenesis is regulated by the hypothalamic–pituitary–testicular axis for the gonadal steroid hormone to occur [2,3]. On the other hand, the testis is endowed with a specialized immune system that becomes tolerogenic towards the antigenic proteins expressed by spermatozoa [4]. Such a characteristic of the testis is known as “immune privilege” in the sense that spermatozoa are protected from autoimmune attacks by gonadal immune cells [5,6]. Furthermore, the testis owns a physical barrier, the so-called blood–testis barrier (BTB), which protects germ cells from noxious immune responses [7]. Alterations of the above-described protective homeostasis by metabolic disorders, infectious events, inflammation and trauma may lead to autoimmunity and infertility [8,9,10].

Microbiota is the assembly of commensal microorganisms located in different districts of the body, which contribute to the health of the host [11]. Particularly, gut microbiota, the most abundant in the body (80%), neutralizes pathogen colonization, exerts metabolic and nutritional activities and takes part in the development of the immune system [12,13,14,15]. Of note, only 9% of the human microbiota is harbored in the urogenital tract, but it is mostly gut microbiota that takes part in male and female sexual maturation. In fact, intestinal metabolites, such as secondary bile acids as well as indole and soybean, regulate male and female sexual organs [16,17,18].

The gut microbiota is composed offour major phyla, i.e., *Bacteroidota, Bacillota, Actinomycetota* and *Pseudomonadota* with *Bacteroidota* and *Bacillota* representing the 90% of intestinal bacteria contingent. The imbalance of gut microbiota, also in relation to high-fat and calorie-rich diets, may lead to a condition of dysbiosis, which with the time canresult in disease status [19]. Moving to the male microbiota, *Lactobacillus*, *Pseudomonas* and *Prevotella* represent the main bacteria contained in the seminal fluid and their replacement by other pathogens may cause dysbiosis, which, in turn, leads to infertility [20].

In this review, special emphasis will be placed on the description of the immune system of the testis, as well as to the components of BTB. Then, the influence of the gut microbiota and male microbiota on the testicular immune system will be illustrated, pointing out all those conditions of dysbiosis which may alter gonadal function and fertility. Novel therapeutic attempts with natural products will also be discussed. All needs must be considered when determining the optimal way to treat an individual patient in the emerging era of personalized medicine.

### 1.1. The Immune Environment of the Testis

**Figure 1:**
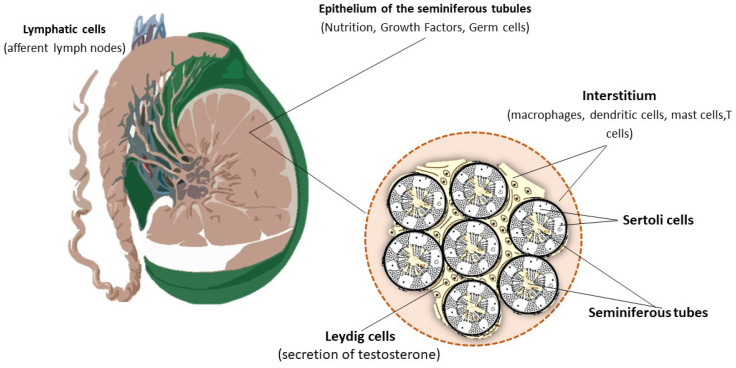
Structure of the testis. The structure of the testis consists of the epithelium of the seminiferous tubules and the intestitium. In turn, the interstium harbors Leydig cells, immune cells and lymphatic vessels.

Immune privilege is confined to a few districts of the body, and, among them, the testicular environment is included. In fact, the proteins expressed on the spermatozoa membrane can elicit a robust immune attack, which may destroy spermatozoa, ultimately leading to infertility. Immune privilege relies on two major mechanisms: (I) the physical shield represented by the BTB, mainly consisting of Sertoli cells (SCs); (II) the tolerogenic response mounted by the immune armamentarium of the testis [21]. In this framework, it is important to briefly describe the structure of the testis for a better comprehension of its function. The epithelium of the seminiferous tubules holds SCs, which provide nutrition and growth factors to germ cells [22,23]. On the other hand, Leydig cells are interspersed between the tubules and secrete testosterone (T) for spermatogenesis to take place [24,25]. The interstitial space of the testis harbors lymphatic vessels, which permit access to afferent lymph nodes [26]. Peritubular lymphatic sinusoids surround the seminiferous tubules with lymphatic capillaries ubicated beneath the tunica albuginea [27]. Macrophages, dendritic cells (DCs), mast cells and T cells are contained in the interstitium, and their function will be described in a specific section of this review. The structure of the testis is depicted in Figure 1.

### 1.2. Blood–Testis Barrier Function

The BTB is constituted by tight junctions (TJs), gap junctions (GJs), desmosome-like junctions and SCs. BTB is maintained by the N-cadherin/beta-catenin of the GJs and occluding/Z0-1 of the TJs all anchored in F-actin bundles [28].Junctions prevent haploid germ cells fromreaching the blood [29]. SCs supply support to germ cells, giving glucose, fatty acids and growth factors, as well asmaintaining an appropriate ionic and metabolic milieu in the testis [30,31]. Moreover, SCs secrete factors which keep an immunoprotected environment in the testis, such as transforming growth factor (TGF)-beta 1 in order to avoid autoimmune destruction of sperm cells [32].

**Figure 2:**
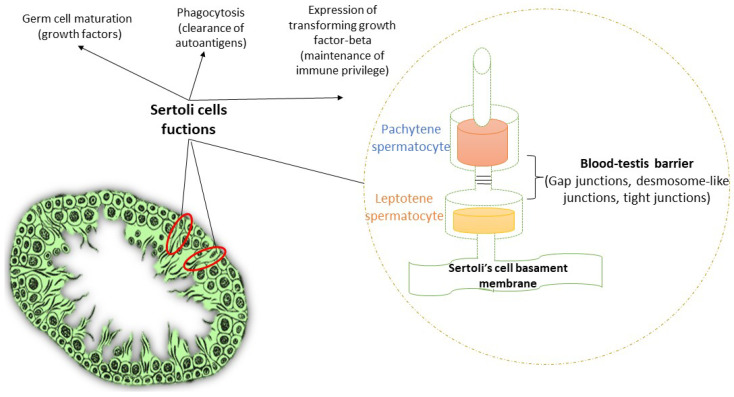
Blood–testis barrier composition. The blood–testis barrier, besides in gap junctions, desmosome-like junctions and tight junctions, has Sertoli cells (SCs). SCs participate in the nutrition and growth of germ cells, maintenance of the immune privilege and clearance of autoantigens and apoptotic cells via phagocytosis.

Another mechanism of protection elicited by SCs is their ability to phagocyte apoptotic germ cells and residual bodies [33]. In order to accomplish their phagocyitc activity, SCs utilize Axl, tyro3 and Mer tyrosine kinase receptors, as well as growth-arrest-specific gene 6 (GAS6). SC-mediated phagocytosis supports spermatogenesis through various mechanisms: (1) making room for the germ cell differentiation process; (2) eliminating harmful substances derived from necrotic germ cells; (3) clearing autoantigens, which may trigger autoimmune responses; (4) providing energy sources to other SCs via the recycle of apoptotic germ cell components [34]. Lastly, SCs switchoff the inflammatory responses of T cells in the testicular interstitium [35]. The BTB is illustrated in Figure 2.

### 1.3. The Immune Arsenal of the Testis

The BTB via SCs constitutes a physical barrier devoted to the protection of germ cells from a destructive immune attack. Besides that, testicular immune cells maintain either a tolerogenic milieu or protect spermatogenesis from pathogen invasion; thus, theycontrol inflammatory processes, which very often are responsible for male infertility [36,37,38]. In fact, evidence has been provided that infections or inflammatory states inhibit steroidogenesis, cause apoptosis of germ cells and destroy testicular epithelial cells, thus provoking infertility [39].

Immune response relies on two major arms, the innate immune system and the adaptive immune system, respectively [40]. Phagocytes(granulocytes and macrophages), natural killer cells and dendritic cells(DCs) [majorantigen-presenting cells (APCs)] are involved in the innate immuneresponse. On the other hand, T and B lymphocytes recognize their specific antigensand maintain immunological memory. Mostly, T cells are divided into differentsubsets, such as T helper (h), T cytotoxic (Tc) and T regulatory (Treg) cells [41].

In the next paragraphs, the functions of testicular macrophages, DCs and lymphocytes will be discussed under both steady state and inflammatory conditions.

*(a)*  *Macrophages*

Testicular macrophages derive from three distinct sources: (1) early yolk sac macrophages; (2) fetal livermonocytes; (3) bone-marrow-derived monocytes [42,43]. Experimental studies have reported that testicular macrophages are able to preserve the local immune privilege, as observed in the testis of rats where these activated phagocytes produce the anti-inflammatory cytokine, interleukin (IL)-10, also expanding T regulatory (Treg) cells [44,45].

Testicular inflammation is caused either by bacteria such as *Escherichia (E.) coli* and *Klebsiella* spp. or viruses (HIV-1, Zika and Mumps orthorubulavirus) [46,47]. Furthermore, in this instance, animal experiments have clarified the detrimental role of infiltrating monocyte-derived macrophages in the promotion of local inflammation, even if the differentiation of peripheral monocytes into testicular macrophages needs further demonstration [48]. Moreover, infected testicular macrophages have been shown to alter SC TJ and interrupt the BTB [49].

Testicular macrophages have been divided into three groups: (1) ED-1 recognizing macrophages, a class of pro-inflammatory cells, which produce tumor necrosis factor-alpha and interferon-gamma; (2) ED-2 macrophages, which exert anti-inflammatory activities by release of IL-10; (3) ED1+ED-2 macrophages, which are a source of nitric oxidase synthase (NOS) [46,47]. ED2+ cells are the majority of the testis macrophages that support a tolerogenic milieu in this organ [50].

*(b)*  *Dendritic Cells*

Dendritic cells (DCs), as professional antigen-presenting cells (APCs), play a tolerogenic effect in the testis, principally leading to Treg cell activation in response to normal sperm antigens [51]. Furthermore, indoleamine 2,3-dioxygenase (IDO), whichcatalyzes the tryptophan metabolism and generates kynurerine, has been found in activated DCs, thus contributing to immune privilege [52]. In fact, kynurerine, acting as a ligand for aryl hydrocarbon receptors on T cells, induces the generation of Foxp3+ Treg cells [51]. Of note, IDO has been shown to induce Treg cell activation in tumors and pregnant uterus, which are also privileged sites, like the testis [53,54].

Under pathological circumstances, in azoospermic humans testicular DCs are able to activate autoreactive T cells, upregulating co-stimulatory molecules, proinflammatory cytokines and major histocompatibility complex class-II (MHC-II), thus leading to male infertility [55,56].

*(c)*  *T Cells*

Treg cells (T cells) have been detected in the mouse, rat and human testis, where they reside in the draining lymph nodes, thus interacting with tissue-specificautoantigens [57]. Located in such a strategic position, Treg cells exert their suppressive function, thus protecting spermatozoa from autoimmune attacks. In this respect, patients with autoimmune regulator gene mutation associated to a defect of Treg cells undergo a chronic testicular inflammation [58]. In chronic inflamed human azoospermic testis, evidence has been provided that Foxp3+ Treg cells are decreased with an increase in the proinflammatory T cell subset, T helper (h) 17 cells [59]. In rat experimental autoimmune orchitis (EAO), CD8+, CD25+, Foxp3+ and CD4+, CD25+, Foxp3+ and Treg cells are increased in the early phase, while the latter subset decreases in the chronic phase [60].

All the above evidence suggests that Treg cells are overly critical in the prevention of organ-specific autoimmunity and maintenance of the immune privilege in the testis. Testicular Th1 cells seem to be necessary for supporting immune homeostasis in this organ. However, an excessive activation of these cells may contribute to autoimmune orchitis [61]. Further studies have proven the intervention of Th17 cells in the later phase of autoimmune orchitis, thus hampering the function of Treg cells, also contributing to the subversion of the testis structure and spermatogenesis [62].

T cytotoxic (c) lymphocytes (CD8+ cells) harbor the testis in a percentage which is 2-fold higher than that of Th cells [63]. Testicular CD8+ cells are functionally associated with resident macrophages or Leydig cells and take part in graft survival [64]. In this respect, pancreatic and islet transplantation in the testis undergoes a lower rate of rejection with an elevated induction of Treg cells [65,66]. This may depend on the SC-mediated activation of Treg cells or on the less potent cytotoxic activity of testicular Tc lymphocytes [67]. To complete the above issue, it is worth mentioning the relationship between T lymphocytes, Leydig cells and SCs, respectively. Leydig cells harbor the interstitial region between seminiferous tubules and represent themajor source of T [68]. Co-cultures of Leydig cells and T cellshave revealed the suppressive effect of the former on the latter, also in view of the binding of Leydig cells to T cells viavascular adhesion molecules [69,70].Androgen receptors are expressed on T cells and, therefore, Leydig cells can modulate their function through androgen secretion.

Experimentally, depletion of T by ethane dimethane sulphonate gives rise to an epididymal sperm granuloma and accumulation of CD4+ and CD8+ T cells, which can be abrogated by supplementation of T [71]. Furthermore, in the EAO rat model, T replacement inhibits the development of autoimmune orchitis through the expansion of Treg cells [72]. Conclusively, Leydig cells are able to limit the infiltration of T cells within the testis, directly and indirectly. SCs are devoted to the protection of spermatogenesis, acting as immunological sentinels. In this regard, it appears that SCs promote the differentiation of tolerogenic DCs and Treg cells [73]. Of note, SCs behave as nonprofessional APCs, expressing MHC-II molecules, thus mediating the expansion of Foxp3+ Treg cells [74].

**Figure 3:**
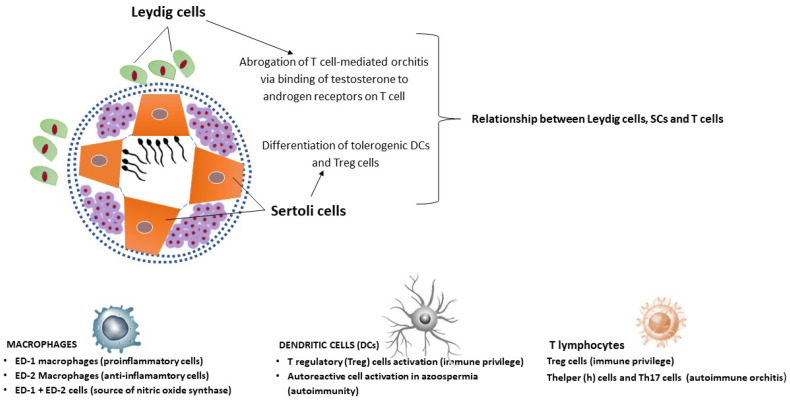
Macrophages, Dendritic cells and T cells sustain the testicular immune response. ED-2 macrophages, DCs and Treg cells keep immune privilege. Conversely, ED-1 macrophages and Th17 cells take part inchronic orchitis progression. Leydig cells attenuate autoimmune orchitis via release oftestosterone. SCs maintain the immune privilege via activation of tolerogenic DCs and Treg cells.

In this direction, transplanted SCs protect syngeneic islet grafts, generating Treg cells and decreasing release of IL-17 by T helper (h)17 cells [75]. In sum, SCs not only contribute to the BTB composition but also keep on check detrimental T cell responses. The testicular immune cells are expressed in Figure 3.

### 1.4. Composition and Function of the Testicular Microbiota

The dogma according to which the testis is an immune privileged site has been contradicted by the evidence that a few bacteria are able to colonize the gonad milieu. In fact, the phyla *Actinomycetota*, *Bacteroidota*, *Bacillota* and *Pseudomonadota* have been detected in testicular biopsies of azoospermic patients [76]. Moreover, the phyla *Bacillota*, *Actinomycetota*, *Bacteroidota* and the genera *Blautia*, *Clostridium* and *Prevotella* were found in testicular specimens of infertile men [77]. In another report, in dyspermic patients and healthy donors *Lactobacillus*, *Pseudomonas*, *Prevotella* and the phyla *Pseudomonadota*, *Bacillota*, *Actinomycetota*, *Bacteroidota* and *Fusobacteria* were identified, with the genus *Prevotella* being inversely associated with sperm concentration, while the *Pseudomonas* genus was correlated with sperm motility [78,79].

Despite the detection of the testicular microbiota, its role in the testis is still debated. According to a recent report, testicular microbiota seems to expand IL-17, producing gamma-delta T cells during puberty, promoting gonadal immune surveillance [80]. It is noteworthy that current research in this specific field has been focused on the link between gut microbiota and testicular microbiota. In the zebrafish model, the genera *Vibrio*, *Aeromonas*, *Pseudomonas* and *Plesiomonas* spp. have been detected in both gut andtestis [81,82]. In the same model, excessive fat intake led to a dramatic reduction of the genus *Vibrio* and *Plesiomonas* spp., with a subversion of signal transduction mechanisms, amino acid transport and metabolism. Furthermore, testicular microbiota regulates the signaling mechanisms of vitamin K and vitamin A and its alteration may change the composition of the extracellular matrix, ultimately leading to male infertility [83,84].

In this direction, evidence has been provided that in a metabolic syndrome model, vitamin A deficit alters the gut–testis axis, finally resulting in an impaired spermatogenesis [85,86]. The gut–testis axis is supported by experimental evidence. Transplantation of fecal flora from high-fat diet (HFD) to normal mice caused an increase in *Bacteroidota* phylum and *Prevotella* genus in normal mice followed by intestinal inflammation and endotoxemia, but mostly by an impaired spermatogenesis [82,87]. In the human counterpart, male infertility is characterized by a negative correlation between *Bacteroidota* phylum and *Prevotella* genus with sperm viability as a result of the “leaky gut hypothesis”. Thus, intestinal endotoxins may impede the T synthesis in Leydig cells, thus provoking a decrease in spermatozoa [88]. More precisely, endotoxins via binding to the TLR-4 expressed on immune cells and epithelial cells can activate the NF-kB pathway with massive release of proinflammatory cytokines [89,90]. In turn, cytokines activate the xanthine oxidase system, thus generating, elevated levels of reactive oxygen species (ROS) and oxidative stress [91].

**Figure 4:**
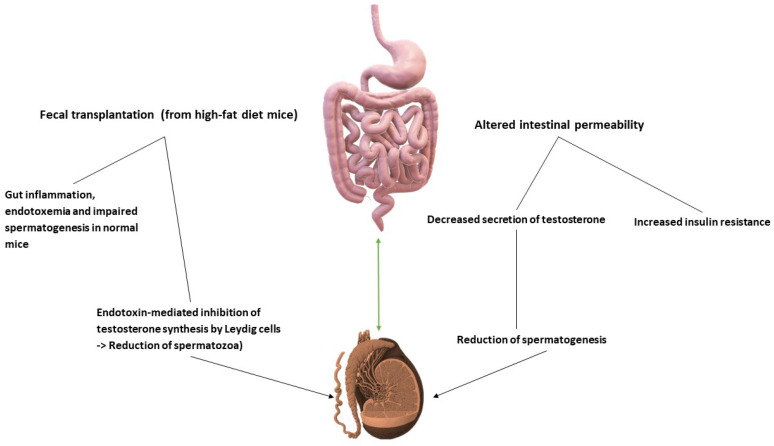
The gut–testis axis. The existence of the gut–testis axis is supported by different evidence. Fecal transplantation from high-fat diet mice to normal mice accounts for endotoxemia occurrence and altered spermatogenesis. In turn, endotoxemia abrogates synthesis of testosterone from Leydig cells, thus reducing the number of spermatozoa with increased release of pro-inflammatory cytokines. Insulin resistance as a result of an altered intestinal permeability leads to a reduced spermatogenesis.

Conclusively, the bacterial translocation-mediated inflammation can account for endothelial damage, subversion of the BTB and alteration of the spermatogenesis and spermatozoa viability [92]. Additionally, DCs and macrophages, which infiltrate the epididymis, are able to capture spermatozoa, thus, contributing, to the impairment of spermatogenesis [93]. Another link between gut microbiota and male reproduction is represented by the endotoxin-mediated insulin resistance (IR), as an expression of altered intestinal permeability [94,95,96]. IR stands for an event of pathogenetic relevance since it alters both gut microbiota and spermatogenesis. In fact, in infertility models with IR, higher levels of *Saccharibacteria* phylum and lower levels of the phyla *Actinomycetota* and *Verrucomicrobia* have been observed in comparison to controls without IR [97]. Parallelly, increased IR is associated with a decreased secretion of T by Leydig cells also in view of a reduced gonadotropin release [98]. In Figure 4 the gut–testis axis is described.

### 1.5. Seminal Dysbiosis with Particular Reference to Male Infertility

The influence of seminal dysbiosis is an issue of current interest. Dyspermic conditions, i.e., oligo-azoospermia, asteno-azoospermia and azoospermia, have been investigated in terms of microbial composition of seminal fluid. For instance, in azoospermic individuals, *Bacteroidota* and *Bacillota* phyla are increased, while the phyla *Pseudomonadota* and *Actinomycetota* are reduced [98]. In the oligo-asteno-teratozoospermic patients, instead, the genera *Neisseria, Klebsiella* and *Pseudomonas* and the phylum *Bacillota* are very abundant, but there is a decrease in *Lactobacillus* [99]. In idiopathic non-obstructive azoospermic patients, the *Clostridium* genus was decreased [100].

Quite interestingly, over the past few years, the influence of female microbiota on the male microbiota has intensively been investigated. For instance, *Gardnerella vaginalis* and the genus *Lactobacillus* have been detected in younger men’s seminal microbiota, while the genera *Pseudomonas, Flavobacterium* and *Acidovorax* have been found in seminal fluid of older individuals [101,102]. On the other hand, inflammatory seminal fluid is associated with *Streptococcus agalactiae*, *Gardnerella vaginalis* and bacterial vaginosis-related bacteria [103].

### 1.6. Microbial-Mediated Male Infertily

Despite the presence of the BTB, the testicular immune arsenal and the local microbiota, the testis can be invaded by urethral pathogens and sexually transmitted bacteria [104]. Acute epididymitis is a very frequent infection of the male reproductive tract, even if this organ has a structure quite overlapping that of the testis [105]. More exactly, epididymis is a less immunologically privileged site in comparison to the testis with a certain degree of immune responsiveness in the caput and an inflammatory profile in the cauda [106,107]. In epididymitis patients, the quality of semen is very low, with an alteration of the protein composition of the sperm, thus contributing to male infertility [108,109]. Persistent pathogen damage leads to fibrotic transformation and epithelial degeneration of the epididymis [110].

Due to the scarcity of human epididymal specimens, research has mainly been conducted on rodent tissue samples. Experimental Gram-negative and Gram-positive infections in the mouse testis have revealed a strong proinflammatory cytokine response with upregulation of NOS-2 [111]. Uropathogenic *Escherichia coli* (UPEC) infections in the mouse are characterized by an activation of TLR4and TLR5 in the epididymis caput with liberation of proinflammatory cytokines and type 1 interferon [112]. On the other hand, epididymal cells respond to UPEC challenge with the production of the antimicrobial peptide defensin b2, which is more effective than gentamycin in reducing bacterial load in both epididymis and testis [113].

**Figure 5:**
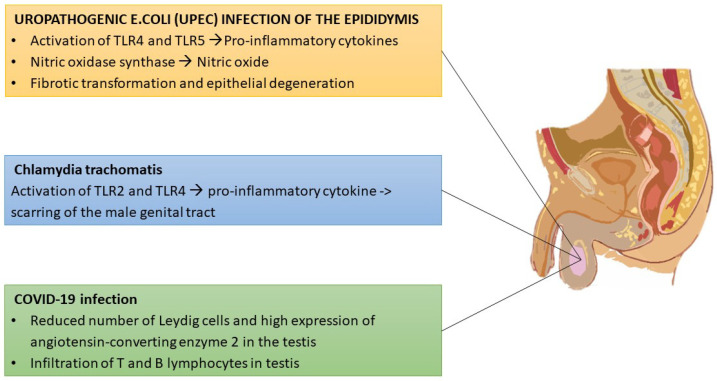
Microbial invasion of the testis. UPEC and *C. trachomatis* damage the male genital tract via production of pro-inflammatory cytokines and nitric oxide, with tissue scarring. COVID-19 infection is characterized by a reduction of Leydig cells, infiltration of T and B cells and elevated expression of ACE-2.

*Chlamydia trachomatis (Ct)* is the most frequent sexually transmitted pathogen in males, leading to chronic inflammation and scarring of the male genital tract [114]. *Ct* antigens bind to TLR2 and TLR4 and pathogen recognition receptors with massive liberation of proinflammatory cytokines, which account for chronicity of inflammation [115]. As far as viral diseases are concerned, mumps virus, an RNA virus, is the most frequent cause of epididymitis and orchitis, which in turn cause male infertility [116]. COVID-19 has been reported to infect the testis, impairing T secretion, thus inducing primary hypogonadism or aggravating a preexistent status of hypogonadism [117]. In particular, a reduced number of Leydig cells have been detected in COVID-19 patients along with a high expression of angiotensin-converting enzyme 2 in the testis [118,119,120,121]. Additionally, involvement of testicular T and B lymphocytes in COVID-19 infection has been reported [122]. Infections of the male reproductive tract are illustrated in Figure 5.

### 1.7. Therapeutic Correction of Testicular Dysbiosis with Natural Products

In view of the connection between microbiota and male reproduction new therapies have been explored. With special reference to personalized medicine, there is a large body of evidence that nutrition can influence the composition of the microbiota, the quality of sperm in terms of caloric content of food components, as well as fatty acid, carbohydrate and protein profiles. In this regard, a high intake of saturated fatty acids may impair male fertility, while a healthy dietary regimen, i.e., the Mediterranean diet (MED) contributes to the preservation of the microbiota and sperm quality [123,124,125]. Conversely, the Western diet causes the rapid spread of obesity associated with hyperinsulinemia and hyperglycemia, which lead to an altered sperm function [126,127].

On these grounds, nutritional interventions to protect male reproduction have been adopted. For instance, MED has been shown to positively affect male reproductive performance, especially through the consumption of extra virgin olive oil (EVOO). According to [128], EVOO is able to change the sperm membrane lipid composition, reducing oxidative stress and enhancing mitochondrial function. Furthermore, MED exerts a homeostatic function in the endocrine–metabolic–immune axis, also shifting the gut microbiota towards an anti-inflammatory profile [129,130]. It is likely that testicular microbiota may be positively affected by MED, but such an assumption needs scientific demonstration. Certain natural products potentially effective in the restoration of testicular microbiota will be illustrated in the following paragraphs.

*(a)*  *Probiotics*

Probiotics by definition are “*live microorganisms which when administered in adequate amounts confer a health benefit on the host*” [131,132]. They have been used to enhance male reproduction, owing to their ability to protect the intestinal barrier, inhibit pathogen growth and activate the immune response [133,134]. In astheno-azoospermic human donors, 3 weeks’ supplementation of *Lactobacillus (L.) rhamnosus* and *Bifidobacterium longum* improved sperm motility while reducing DNA fragmentation [135,136]. In another study, administration of a symbiotic, Familact^®^, composed by *Lactobacillus* strains and oligo-fructosaccharides, to idiopathic male infertility could enhance sperm quality and DNA integrity, while reducing free radicals in the semen [137].

Experimentally, in HFD obese mice, supplementation of *L. rhamnosus* improved spermatozoa motility, increasing the number of Leydig cells [138]. In infertile mice, administration of *Lactobacillus* spp., *Bacillus* spp., *Saccharomyces cerevisiae* (beer yeast) and photosynthetic bacteria cultures reduced sperm damage and improved motility [139].

*(b)*  *Prebiotics*

Oligofructose, galacto-oligosaccharides and breast-milk oligosaccharides are the most representative prebiotics, endowed with the ability to increase levels of *Bifidobacterium* and *Lactobacillus*, as well as of SCFAs [140,141]. In a preclinical study, evidence has been provided that manno-oligosaccharides were able to accelerate sexual maturation in rats [142]. In particular, the decrease in blood corticosterone observed in this study could account for the elevated levels of T and maturation of seminiferous tubules. To the best of our knowledge, no clinical trials have been conducted to treat male infertility with prebiotics.

*(c)*  *Antioxidants*

Vitamin C and vitamin E can exert especially beneficial effects in infertile men, reducing ROS levels, improving sperm motility and maintaining DNA integrity [143]. Among other antioxidants, lycopene, present in tomatoes and red fruits, seems to display positive effects on the testicular mitochondria by modulating lipid peroxidation within the mitochondrial membrane [144]. Conversely, other studies based on theadministration of antioxidants did not show any improvement of semen biomarkers and DNA integrity in infertile men [145,146].

*(d)*  *Polyphenols*

Polyphenols are natural compounds, mainly contained in fruits, vegetables, oil, wine and cocoa [147,148]. They exert potent anti-inflammatory and antioxidant activities on different cell types, even including spermcells [149,150,151]. Experimental and human studies have been undertaken with quercetin and resveratrol; however, results have been quite controversial, since both polyphenols are endowed with antioxidant and pro-oxidant activities [152,153].

Table 1 shows the main natural products putatively involved in the treatment of male genital tract infections.

## 2. Conclusions

A mutual cooperation between testicular immunity and microbiota contribute to normal spermatogenesis and sperm maturation. Such an equilibrium may be subverted by a range of factors, even including concurrent pathologies, e.g., obesity, diabetes, infections and trauma. Among novel therapeutic approaches to restore male infertility, a proper nutritional regimen, as in the case of MED, may be useful in male infertility associated to obesity and diabetes. Furthermore, supplementation of natural products, such as probiotics, prebiotics, antioxidants and polyphenols has been demonstrated to enhance male reproductive function either in animal models or clinical trials. However, also, in view of conflicting results, more clinical attempts are needed to establish the actual effectiveness of natural products for the correction of testicular microbiota and immune function in the era of personalized medicine.

## Figures and Tables

**Table 1:** Natural products for the correction of testicular dysbiosis. Both probiotics and synbiotics are able to improve sperm quality and motility in male infertility. In rats, manno-oligosaccharides (prebiotics) promote sexual maturity. Polyphenols exert both antioxidant and pro-oxidant activities. Among antioxidants, vitamin C and vitamin E are able to reduce ROS generation and improve sperm mobility and DNA integrity. Lycopene enhances sperm performance via lipid peroxidation on mitochondrial membranes.

Natural Products against Testicular Dysbiosis			
PROBIOTICS	PREBIOTICS	POLYPHENOLS	ANTI-OXIDANTS
In astenoazoospermic human donors In idiopathic male infertility symbiotic-induced Lactobacillus-mediated improvement of enhancement of sperm quality and reduction of sperm motility and DNA fragmentation free radicals in the semen	Mann-oligosaccharides acceleration of sexual maturity in rats	Controversial results with quercetin and resveratrol since they are endowed with both anti-oxidants and pro-oxidants activities	Vitamin C and vitamin E -> reduction of ROS and improvement of sperm mobility and DNA integrity Lycopene -> modulation of lipid peroxidation on mitochondrial membrane

## Data Availability

Data are contained within the article.
